# Molecular clustering of patients with diabetes and pulmonary tuberculosis: A systematic review and meta-analysis

**DOI:** 10.1371/journal.pone.0184675

**Published:** 2017-09-13

**Authors:** Francles Blanco-Guillot, Guadalupe Delgado-Sánchez, Norma Mongua-Rodríguez, Pablo Cruz-Hervert, Leticia Ferreyra-Reyes, Elizabeth Ferreira-Guerrero, Mercedes Yanes-Lane, Rogelio Montero-Campos, Miriam Bobadilla-del-Valle, Pedro Torres-González, Alfredo Ponce-de-León, José Sifuentes-Osornio, Lourdes Garcia-Garcia

**Affiliations:** 1 Doctorado en Ciencias en Enfermedades Infecciosas, Centro de Investigación sobre Enfermedades Infecciosas, Instituto Nacional de Salud Pública, Cuernavaca, Morelos, México; 2 Centro de Investigación sobre Enfermedades Infecciosas, Instituto Nacional de Salud Pública, Cuernavaca, Morelos, México; 3 Maestría en Ciencias Médicas con énfasis en Epidemiología, Facultad de Medicina, Universidad Nacional Autónoma de México, Distrito Federal, México; 4 Facultad de Medicina, Universidad Autónoma de San Luis Potosí, San Luis Potosí, San Luis Potosí, México; 5 Laboratorio de Microbiología, Instituto Nacional de Ciencias Médicas y Nutrición “Salvador Zubirán”, México, Distrito Federal, México; 6 Dirección Médica, Instituto Nacional de Ciencias Médicas y Nutrición “Salvador Zubirán”, México, Distrito Federal, México; University of Cape Town, SOUTH AFRICA

## Abstract

**Introduction:**

Many studies have explored the relationship between diabetes mellitus (DM) and tuberculosis (TB) demonstrating increased risk of TB among patients with DM and poor prognosis of patients suffering from the association of DM/TB. Owing to a paucity of studies addressing this question, it remains unclear whether patients with DM and TB are more likely than TB patients without DM to be grouped into molecular clusters defined according to the genotype of the infecting *Mycobacterium tuberculosis* bacillus. That is, whether there is convincing molecular epidemiological evidence for TB transmission among DM patients. Objective: We performed a systematic review and meta-analysis to quantitatively evaluate the propensity for patients with DM and pulmonary TB (PTB) to cluster according to the genotype of the infecting *M*. *tuberculosis* bacillus.

**Materials and methods:**

We conducted a systematic search in MEDLINE and LILACS from 1990 to June, 2016 with the following combinations of key words “tuberculosis AND transmission” OR “tuberculosis diabetes mellitus” OR “*Mycobacterium tuberculosis* molecular epidemiology” OR “RFLP-IS6110” OR “Spoligotyping” OR “MIRU-VNTR”. Studies were included if they met the following criteria: (i) studies based on populations from defined geographical areas; (ii) use of genotyping by IS6110- restriction fragment length polymorphism (RFLP) analysis and spoligotyping or mycobacterial interspersed repetitive unit-variable number of tandem repeats (MIRU-VNTR) or other amplification methods to identify molecular clustering; (iii) genotyping and analysis of 50 or more cases of PTB; (iv) study duration of 11 months or more; (v) identification of quantitative risk factors for molecular clustering including DM; (vi) > 60% coverage of the study population; and (vii) patients with PTB confirmed bacteriologically. The exclusion criteria were: (i) Extrapulmonary TB; (ii) TB caused by nontuberculous mycobacteria; (iii) patients with PTB and HIV; (iv) pediatric PTB patients; (v) TB in closed environments (e.g. prisons, elderly homes, etc.); (vi) diabetes insipidus and (vii) outbreak reports. Hartung-Knapp-Sidik-Jonkman method was used to estimate the odds ratio (OR) of the association between DM with molecular clustering of cases with TB. In order to evaluate the degree of heterogeneity a statistical Q test was done. The publication bias was examined with Begg and Egger tests. Review Manager 5.3.5 CMA v.3 and Biostat and Software package R were used.

**Results:**

Selection criteria were met by six articles which included 4076 patients with PTB of which 13% had DM. Twenty seven percent of the cases were clustered. The majority of cases (48%) were reported in a study in China with 31% clustering. The highest incidence of TB occurred in two studies from China. The global OR for molecular clustering was 0.84 (IC 95% 0.40–1.72). The heterogeneity between studies was moderate (I^2^ = 55%, p = 0.05), although there was no publication bias (Beggs test p = 0.353 and Eggers p = 0.429).

**Conclusion:**

There were very few studies meeting our selection criteria. The wide confidence interval indicates that there is not enough evidence to draw conclusions about the association. Clustering of patients with DM in TB transmission chains should be investigated in areas where both diseases are prevalent and focus on specific contexts.

## Introduction

Tuberculosis (TB) remains one of the main causes of morbidity and mortality in low- and medium-income countries, where the number of individuals with diabetes mellitus (DM) is rapidly increasing.

For more than 30 years genotypic analyses of *Mycobacterium tuberculosis* together with conventional epidemiologic methods have helped to further characterize *M*. *tuberculosis* strains [1–3] and understand the dynamics of transmission of TB in different regions and populations [4–7]. Patients with identical strains of *M*. *tuberculosis* are considered to belong to molecular clusters, their disease being due to recent transmission and rapid progression. Unique genetic patterns are likely due to reactivation of latent infection or recent transmission from patients out of the period or area under study [6, 7].

A great variety of individual, biological and social determinants have been associated with clustering of pulmonary TB (PTB) cases. In highly endemic areas for TB some of these factors include being male, young, having been born in an endemic country, resident of an urban area, alcohol or drug consumption, homelessness, HIV infection or having acid-fast bacilli in sputum smear [6, 8–11]. Similar determinants have been described in medium income countries—young age, previous imprisonment and visits to social environments [12]. There are conflicting results on the association of HIV associated immunodeficiency and increased risk of transmission of *M*. *tuberculosis* in African countries where both diseases are highly prevalent [13–15]. Fewer studies have investigated if DM increases the likelihood of PTB patients to cluster according to the genotype of the infecting *M*. *tuberculosis* bacillus.

Owing to a paucity of studies addressing this question, it remains unclear whether patients with DM and TB are more likely than TB patients without DM to be grouped into molecular clusters defined according to the genotype of the infecting *M*. *tuberculosis* bacillus. That is, whether there is convincing molecular epidemiological evidence for TB transmission among DM patients. This information would contribute to developing effective preventive strategies. We conducted a systematic review and meta-analysis to quantitatively evaluate the propensity of patients with DM and PTB to cluster according to the genotype of the infecting *M*. *tuberculosis* bacillus.

## Materials and methods

We conducted this study according to the Meta-analysis of Observational Studies in Epidemiology (MOOSE) guidelines [16] and the Preferred Reporting Items for Systematic Reviews and Meta-Analyses guidelines [17], S1 Table.

### Search strategy

A systematic search was made in the electronic data bases of MEDLINE and LILACS, from 1990 to June, 2016. The following combinations of key words were used: "tuberculosis AND transmission" OR "tuberculosis AND diabetes mellitus” OR “*Mycobacterium tuberculosis* molecular epidemiology” OR “RFLP-IS6110” OR “Spoligotyping” OR “MIRU-VNTR”. Two independent reviewers selected potentially relevant articles based on the title and abstracts. We included publications in English or Spanish reporting original data from observational studies in which DM was evaluated among other risk factors for molecular clustering of PTB cases. We contacted the authors of selected articles to clarify methodological aspects and results.

Studies were included if they met the following criteria: (i) studies based on populations from defined geographical areas; (ii) use of genotyping by IS6110- restriction fragment length polymorphism (RFLP) analysis and spoligotyping or mycobacterial interspersed repetitive unit-variable number of tandem repeats (MIRU-VNTR) or other amplification methods to identify molecular clustering; (iii) genotyping and analysis of 50 or more cases of PTB; (iv) study duration of 11 months or more; (v) identification of quantitative risk factors for molecular clustering including DM; (vi) > 60% coverage of the study population; and (vii) patients with PTB confirmed bacteriologically. The exclusion criteria were: (i) Extrapulmonary TB; (ii) TB caused by nontuberculous mycobacteria; (iii) patients with PTB and HIV; (iv) pediatric PTB patients; (v) TB in closed environments (e.g. prisons, elderly homes, etc.); (vi) diabetes insipidus and (vii) outbreak reports. References lists of all included studies were screened to identify further potentially eligible studies.

### Data extraction

Titles, abstracts, and full-text articles identified from the searches were screened by one reviewer (FBG) to select relevant articles. A second reviewer (GDS) independently screened 10% of titles and abstracts and 30% of full-text articles. Two authors (FBG and GDS) independently extracted data from included studies using previously piloted data extraction forms. The extracted information included: study design, region, period, number of genotyped subjects, sampling fraction, diagnostic criteria for DM, genotyping method, definition of molecular clustering, proportion of cases in the clusters, definition of index case and contact tracing. If secondary genotyping was used, the combined methods as reported by the authors were reported. When TB rates were not available in the citation, we used data from the World Bank [18]. Disagreements and discrepancies in study selection and data extraction were resolved through discussion.

### Statistical analysis

The statistical analysis was based on a model of random effects to estimate the odds ratio (OR) (Mantel-Haenszel) of DM associated with molecular clustering of PTB. Pooled OR and 95% Confidence intervals (95% CI) of the association between DM and molecular clustering were estimated using random effect meta-analyses with the Hartung-Knapp-Sidik-Jonkman modification. We used the Hartung-Knapp-Sidik-Jonkman approach since is a more robust method for meta-analyses with moderate heterogeneity (>50%), few studies (between 5 and 20) and varied sample sizes as compared to DerSimonian and Laird method.[19] Heterogeneity between the studies was assessed using the *I*^2^ statistic [20].

Patients with PTB with the same genotype were classified as clustered and those with unique genotypes as not clustered.

#### Sensitivity analysis

To identify the influence of the Borrell et al. [21], we repeated the meta-analysis without this study [22, 23] using the Hartung-Knapp-Sidik-Jonkman test.

#### Bias evaluation

We conducted bias evaluation based on the Cochrane manual [24]. Because evaluation was heterogeneic and, following the manual´s recommendations, we conducted an evaluation of publication bias.

#### Evaluation of publication bias

Because the presence of publication bias can lead to incorrect estimations and false conclusions that affect the validity of the meta-analysis of observational studies, publication bias was analyzed using Beggs tests [25] and Egger [22]. The sample size of each study was compared with the size of the observed effect. A p value of <0.10 was considered statistically significant [23].

Statistical analysis was performed using Review Manager 5.3.5 (Rev Man for Windows, 2015; The Cochrane Collaboration, Oxford, United Kingdom) and Comprehensive Meta-Analysis 3 (CMA v.3, Biostat) [26] and Biostat and Software package R were used.

## Results

**Fig 1:**
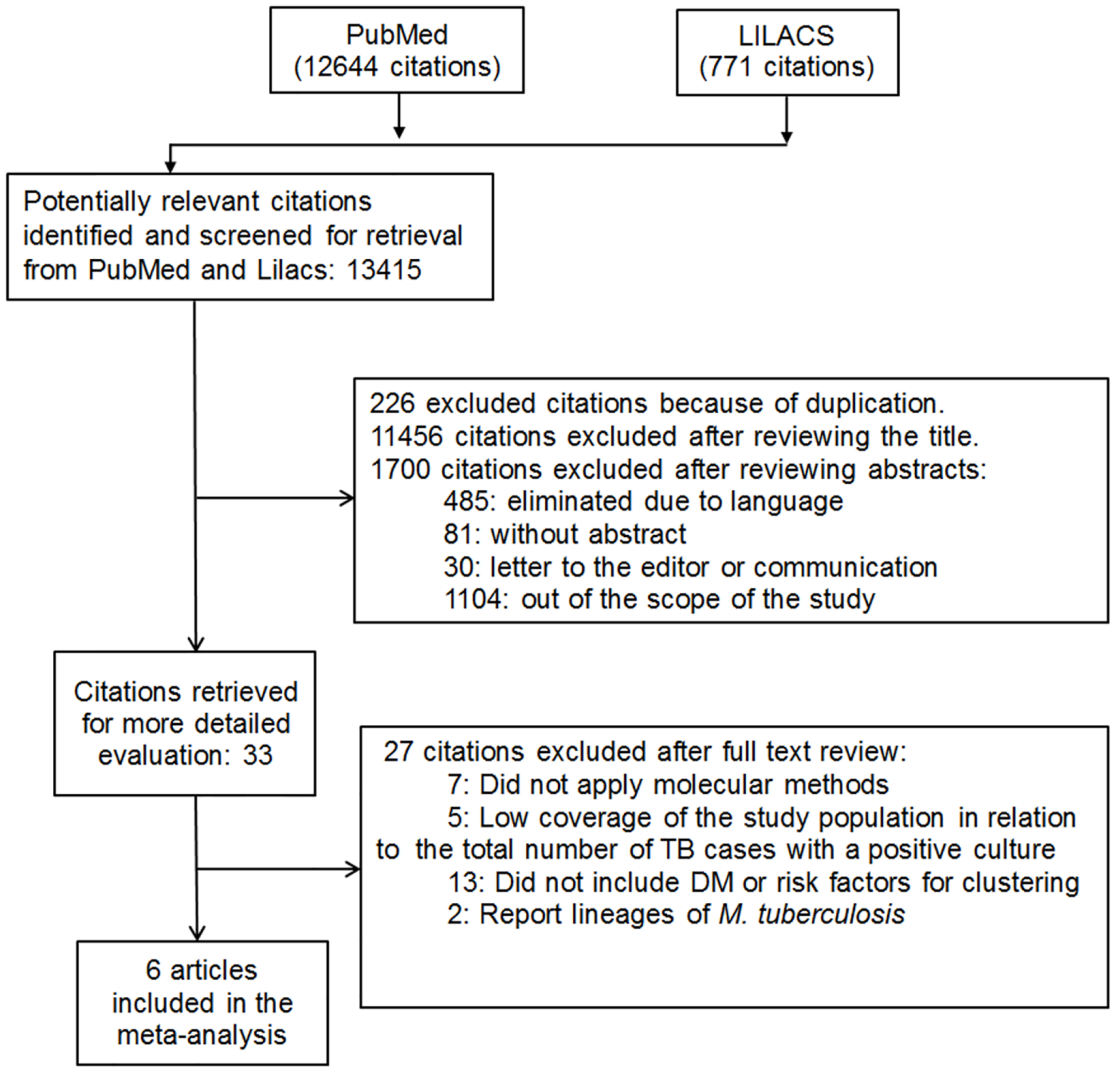
Flow diagram of electronic search.

The electronic search produced a total of 13415 articles of which 226 were excluded for duplicity leaving 13189 potentially relevant studies. After a title and abstract revision, 13156 articles were excluded for various reasons: (i) language different to English or Spanish, (ii) had no abstract, (iii) letter to the editor or communication, (iv) outside the scope of this study. After full reading of the 33 articles, 27 were excluded for the following reasons: (i) seven studies [27–33] did not use molecular methods for the characterization, (ii) five studies [34–38] had low coverage of the studied population in regard to the total number of patients with a positive culture for TB, (iii) 13 studies [39–51] did not show the quantification of risk factors for clustering or did not include DM and (iv) two studies [52, 53] reported only the genetic lineages of *M*. *tuberculosis*. Finally six articles [21, 54–58] met all the inclusion criteria for the quantitative analysis. Fig 1 provides a flow diagram of the systematic selection of the literature.

The literature search for studies on the association between DM and molecular clustering of strains of patients with PTB.

The general characteristics of the selected articles are summarized in Table 1. All six studies were cohort studies, two were conducted in China [57, 58] and one in Spain [21], Canada [55], Mexico [56] and Cuba [54]. Among the 6 studies there were 4076 patients with PTB of which 13% had comorbidity with DM. The total percentage of clustering was 27%. Percentage clustering was similar among patients with DM (25%) and without DM (28%). The majority of cases (48%) were reported by a Chinese study [58] with 31% clustering. The highest annual incidence of TB was reported by the studies from China (73/100,000 inhabitants).

**Table 1:** General characteristics of the studies.

Author and year of publication	Study region	Study period	Design of study	TB annual incidence rate per 100 000 inhabitants	Study duration (months)	Number of genotyped subjects	Sampling fraction (%)	DM diagnosis based on:	Primary genotyping method	Secondary genotyping method	Application of secondary genotyping method	Cluster definition	Index case of the cluster	Subjects in the cluster (%)	Contact tracing
Hernández-Garduño et al., 2002 [55].	Greater Vancouver, British Columbia, Canada	1995–1999	Cohort	6.0**	50	793	100	Information obtained from the dataset of the TB division.	RFLP-IS6110	Spoligotyping	< 6 copies of IS6110	≥ 2 strains with identical DNA (≥ 6 copies with RFLP, or < 6 copies with s poligotyping	First diagnosed patient	17.3	Yes
Borrell et al., 2010 [21].*	Barcelona, Spain	2003–2004	Cohort	21.6	24	115	67.4	Information obtained from TB program dataset of Barcelona and microbiology areas of hospitals and clinical centers.	RFLP-IS6110	MIRU-12 loci	< 6 copies of IS6110 or ≥6 bands with a different one	≥ 2 patients: (i) RFLP-IS6110 ≥ 6 bands in the same position; (ii) RFLP-IS6110 < 6 bands in the same position but with identical MIRU-12 loci; (iii) RFLP-IS6110 ≥ 6 bands that differ in 1 band but with identical MIRU-12 loci	Subject with earliest onset of pulmonary symptoms or the one that started treatment first (asymptomatic)	32	Unknown
Jiménez-Corona et al., 2013 [56].	Orizaba, Veracruz, México	1995–2010	Cohort	21**	181	1013	80	Previous diagnosis from a physician or oral hypoglycemic medication or insulin administration or treatment	RFLP-IS6110	Spoligotyping	< 6 copies of IS6110	≥ 2 two or more isolates from different patients identified within 12 months of each other and with six or more IS6110 bands in an identical pattern, or < 6 bands with identical IS6110 RFLP patterns and a spoligotype with the same spacer oligonucleotides	Not specified	28.8	Unknown
Wang et al., 2014 [57].	Beijing, China	2009–2011	Cohort	73**	48	115	100	Clinical data obtained from the patient`s medical history	MIRU-VNTR 24 loci (up to 28)	Does not apply	Does not apply	≥ 2 strains showing identical patterns MIRU-VNTR	Not specified	17.4	Unknown
González et al., 2015 [54].	La Habana, Cuba	2009	Cohort	8.2	12	59	61	Clinical information obtained from the national statistics / epidemiology data base of the public health ministry of Cuba	MIRU-VNTR 24 loci	Does not apply	Does not apply	Strains with identical genetic pattern	Not specified	54.2	Yes
Yang et al., 2015 [58].	China	2009–2012	Cohort	73**	36	1948	82	Clinical information obtained by professional interviewers with standardized questionnaire.	MIRU-VNTR 29 loci	Does not apply	Does not apply	Strains with identical genetic pattern	First patient in the cluster	31	Yes

*We used only information from foreign patients;

** Annual rates were taken from World Bank [18]

Diagnosis of DM was obtained from various sources: data collection formats [53, 54], TB control program or statistical center [55, 57] and patient’s medical history [21]. Only one study [56] described diagnostic criteria for DM.

The genotyping methods used in the studies were IS6110-RFLP analysis and spoligotyping and MIRU-VNTR. Therefore, definitions of molecular clustering and method of identification of the index case differed between studies.

**Fig 2:**
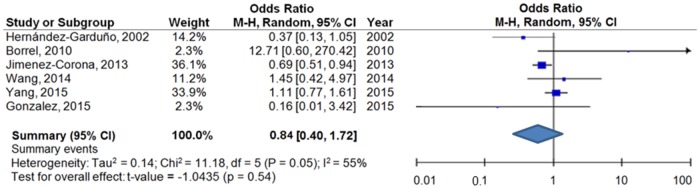
Molecular clustering risk of patients with PTB and DM compared with patients with PTB.

The pooled OR for molecular clustering of patients with DM and TB compared to patients with TB was 0.84 (CI 95% 0.40–1.72) with the Hartung-Knapp-Sidik-Jonkman test (Fig 2). There was a moderate heterogeneity [24] between studies (I^2^ = 55%, p = 0.05) (Fig 2).

We did not find a statistically significant publication bias (p<0.1) according to Beggs (p = 0.353) and Egger tests (p = 0.429).

Size of the square is proportional to the precision of the study-specific effect estimates, and the bars indicate the corresponding 95% confidence intervals (CIs). Odds ratio (OR) was calculated using the OR, CI and total number of patients with and without DM provided in the paper. Pooled OR and 95% CI of the association between DM and molecular clustering were estimated using random effect meta-analyses with the Hartung-Knapp-Sidik-Jonkman modification.

When we excluded Borrell et.al.[21] study, the pooled OR for molecular clustering of patients with DM and TB compared to patients with TB was 0.79 (CI 95% 0.42–1.748), similar to our previous result, (S1 Fig).

## Discussion

This systematic review and meta-analysis included 6 cohort studies published between 2002 and 2015 that comprised a total of 4076 patients with PTB of which 13% also had DM. The meta-analysis did not show an association between DM and molecular clustering of PTB in the community. The pooled estimate was 0.84 (CI 95% 0.40–1.72). The wide confidence interval indicates that there is not enough evidence to draw conclusions about the association.

Many studies have explored the relationship between DM and TB, including a recent systematic review demonstrating that the risk of TB among people with DM triples that of people without DM [59] while other studies show poorer outcomes among patients with TB and DM [34, 56, 59–65]. Public health impact of the comorbidity is greater in regions of low and middle income, where 84% of patients with DM live, many of whom are unaware of their condition. [66, 67]. In fact, an estimate of incident TB cases attributable to DM increased from 10% to 15% from 2010 to 2013 in the 22 countries with 80% of the global burden of TB [67].

Molecular tools have provided direct evidence for the occurrence of exogenous reinfection among both immunocompetent and immunocompromised individuals [68]. As the present review shows, patients with DM have not been specifically studied [69, 70]. Clinical manifestations among patients with TB and DM such as delayed sputum and culture conversion [56, 63, 71], higher likelihood of pulmonary (versus extra pulmonary) forms [72] and cavitation [56] due to dysfunctional innate and acquired immune system indicate that patients with DM might have an important role in TB transmission. In addition, patients with DM and hyperglycemia have a greater risk of infection and disease [73], which would increase their likelihood of participating in chains of transmission. Furthermore usage of molecular epidemiologic techniques has previously allowed us to show that increased risk of TB among patients with DM is due to both reactivation and recently transmitted infection [62]. More recently, we demonstrated that patients with DM who presented with a subsequent TB episode, were more likely to suffer from infections caused by the same bacteria as the previous episode [56]. However, the occurrence of exogenous reinfection in one-fifth of the cases suggested that exogenous TB reinfection in DM patients might be due to nosocomial TB transmission occurring as a result of DM patients attending clinics where there is a high prevalence of diagnosed and undiagnosed TB, as has been described for HIV infected patients [74]. Patients with DM have been described as index and secondary cases in TB outbreaks [75, 76]. Therefore, there are grounds to hypothesize that patients with DM and TBP may have a greater likelihood of recent transmission than TBP patients without DM.

Recently, a large study of recent transmission in the United States reported substantial geographic heterogeneity in the proportion of cases attributed to recent transmission [77]. Patient characteristics associated to recent transmission differed according to cluster size. Therefore, study of characteristics associated to TB clustering is complex and probably specific to each setting.

The rise in TB incidence in persons with DM seems to have multiple causes. Although the physiopathology of TB susceptibility in patients with DM remains to be clarified, changes in the immune system have been described, including alterations in the complement pathway in patients with DM [78], increase in type 1 innate cytokines [79, 80], a reduction in the activation of alveolar macrophages [81], and increased IL-10 producing ability [82, 83]. Other authors have reported a reduction in Th1 cytokines [84]. Based on murine experiments, it has been suggested that TB susceptibility in DM can cause a delay in the initiation and expression of adaptive immunity [85]. In a recent review, the authors suggested that the interaction between the host and *M*. *tuberculosis* can be explained by the weakening of innate immunity followed by a hyper-reactive cell response [86]. Therefore, DM deteriorates cell mediated immunity by altering the function and activation of macrophages, monocytes and lymphocytes with possible potential roles for pulmonary microangiopathy, renal dysfunction and vitamin deficiencies [63, 64, 87]. In addition, patients with sustained hyperglycemia seem to be at a higher risk of acquiring TB than those with controlled blood sugar levels suggesting that hyperglycemia is an important determinant in this interaction [63, 64, 87–89].

Association of TB with chronic diseases has been scarcely studied with the exception of TB and HIV/AIDS. Studies have provided conflicting results regarding the association between HIV infection and recent transmission of TB [13–15, 77]. The lack of association could be partially explained by the decreased risk of exposure to TB in patients with HIV in regions where incidence rates of TB have decreased or adequate antiretroviral coverage has been achieved [77, 90], although it has also been reported that antiretroviral treatment does not give protection against TB progression after a recent infection [15]. Clinical characteristics of TB among HIV patients such as degree of immunosuppression, less bacillary load, and decreased frequency of cavitary disease may also impact over infectiousness [14]. In contrast, it has been found that HIV infected patients are more likely to aggregate in molecular clusters suggesting that HIV infection increases an individual’s risk of TB disease due to recent *M*. *tuberculosis* infection [13].

### Limitations

This study is limited by the lack of a general agreement over a recognized methodological standard in conducting population-based TB molecular epidemiological studies. Multiple characteristics impact over clustering frequency including TB incidence, study duration, intensity of contact tracing, migration patterns into the study area, size of clusters, sampling fraction, occurrence of endemic strains, frequency of strains with low copy numbers, and age of study populations [66]. We adjusted for some of these factors through our selection criteria (study design, sample fraction, number of participants, and study duration). Our strict inclusion criteria partly explain the small number of studies that were included in the study. There has been extensive discussion in the literature of the validity of estimating pooled effects when the systematic review results in a small number of studies. We agree with Valentine *et*. *al*. who recommend that doing the meta-analysis with few studies allows the reviewers to compute and interpret confidence intervals and as such add information beyond what is revealed by the individual studies.[91] Furthermore, numbers of studies eligible for meta-analyses are typically very small for all medical areas as was shown in a study of meta-analyses and their component studies in the Cochrane Database of Systematic Reviews (the median number of studies was 3 (IQR 2–6).[92] Therefore, our study is above the median of number of studies included in meta-analyses in this highly recognized data set. Another possible bias might have occurred if sources of DM diagnosis were different. We consider this unlikely since data sources were clinical or public health datasets. The articles included in this meta-analysis based their genotypic characterization on the two most frequently reported methods-RFLP-IS6110 and MIRU-VNTR. RFLP and VNTR methods have been widely recognized as tools with similar if not equal power when determining the epidemiologic relationship between M. tuberculosis strains [93–97]. A TB control program pioneer in the use of RFLP-IS6110 in operational conditions, showed an overall concordance in clustering of 79% and highly similar discriminatory power in the two methods when typing 3,978 M. tuberculosis isolates [97]. Our study is also limited by the fact that reviewed studies did not consider the increasing evidence of within patient microdiversity arising both from “de novo” mutations and mixed infections [98, 99]. As a consequence, samples taken from the upper airway (as occurred in the majority of included studies in this review) captured only a fraction of the bacterial population diversity. Future molecular epidemiological studies will need to consider microdiversity and mixed infections since they challenge current understanding of transmission dynamics.

Finally, we excluded studies of patients with PTB and HIV, therefore not considering patients that may have suffered from PTB, HIV and DM and limiting the generalizability of our results particularly to southern Africa where TB and HIV are highly prevalent. The International Diabetes Federation estimates that DM prevalence in Africa is 3.8% (2.6–7.9%) with over two thirds (66.7%) of people with diabetes unaware they have the disease [100]. Moreover, the availability of antiretroviral treatments has extended survival and ageing, with concomitant increase in non-communicable diseases, including DM. However, there is limited knowledge on the association between DM and TB in HIV-infected people from sub-Saharan Africa [101]. Therefore, we would have missed very few molecular epidemiological studies describing the triple morbidity.

### Conclusion

Clustering of patients with DM in TB transmission chains should be investigated in areas or countries with high TB and DM rates. Studies with the purpose of better understanding the demographic, clinical, social and geospatial factors associated with TB in regions with a high comorbidity with DM should be conducted to better understand transmission dynamics as has recently been proposed for the association of TB and HIV[102]. Research needs to focus on specific settings investigating if TB transmission is more likely to occur in meeting points such as clinical facilities, clubs, camps, religious gatherings, etc. particularly in countries highly endemic for both diseases.

## Supporting information

S1 Table Prisma checklist. (DOC)

S1 Fig Molecular clustering risk of patients with PTB and DM compared with patients with PTB excluding Borrell et.al.[21]. Size of the square is proportional to the precision of the study-specific effect estimates, and the bars indicate the corresponding 95% confidence intervals (CIs). Odds ratio (OR) was calculated using the OR, CI and total number of patients with and without DM provided in the paper. Pooled OR and 95% CI of the association between DM and molecular clustering were estimated using random effect meta-analyses with the Hartung-Knapp-Sidik-Jonkman modification.
(TIF)
