# Supplementary figures and images for: Molecular clustering of patients with diabetes and pulmonary tuberculosis: A systematic review and meta-analysis
Source: PLoS One. 2017 Sep 13;12(9):e0184675. doi: 10.1371/journal.pone.0184675 (PMC5597214; doi:10.1371/journal.pone.0184675)

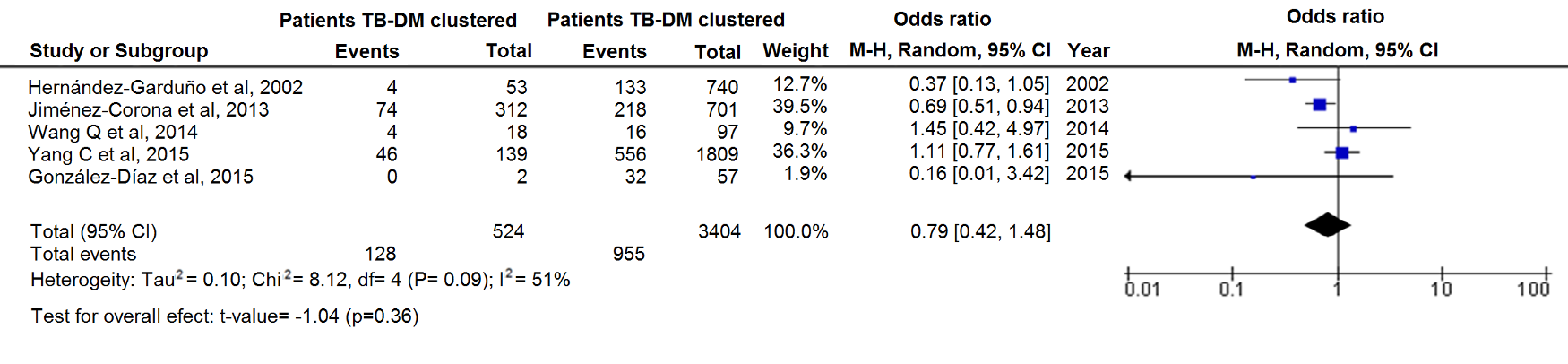

Supplement: S1 Fig — Size of the square is proportional to the precision of the study-specific effect estimates, and the bars indicate the corresponding 95% confidence intervals (CIs). Odds ratio (OR) was calculated using the OR, CI and total number of patients with and without DM provided in the paper. Pooled OR and 95% CI of the association between DM and molecular clustering were estimated using random effect meta-analyses with the Hartung-Knapp-Sidik-Jonkman modification. (TIF) [file pone.0184675.s002.tif]
